# Time Point, Etiology, and Short-Term Outcome of Repeated Mechanical Thrombectomy Due to Recurrent Large Vessel Occlusion

**DOI:** 10.3389/fneur.2019.00204

**Published:** 2019-03-08

**Authors:** Ralph Weber, Paul Stracke, René Chapot

**Affiliations:** ^1^Department of Neurology, Alfried Krupp Krankenhaus, Essen, Germany; ^2^Faculty of Medicine, Ruhr-University Bochum, Bochum, Germany; ^3^Department of Radiology and Neuroradiology, Alfried Krupp Krankenhaus, Essen, Germany

**Keywords:** ischemic stroke, mechanical thrombectomy, recurrence, large vessel occlusion, time point

## Abstract

**Background:** Data on time point and etiology of repeated stroke caused by large brain vessel occlusion (LVO) resulting in repeated mechanical thrombectomy (MT) in acute stroke patients is very limited.

**Methods:** We retrospectively reviewed all acute stroke patients treated with MT with stent retrievers or aspiration systems between January 2010 and June 2018 to identify patients who received two or more MT treatments due to recurrent LVO in our tertiary neurovascular center. Short-term outcome was assessed using the NIH stroke scale score at discharge.

**Results:** We identified 35 out of 2,470 acute stroke patients treated with MT who had recurrent LVO and received repeated MT. Mean age at first MT was 69.3 (±15.8) years and the mean time interval between the first and second MT was 147 (±241) days, and 8 patients experienced short-term LVO recurrence within 3 days. Fifteen (43%) patients had cardioembolic, 9 (26%) arterioembolic, 4 (11%) mixed cardio-/arterioembolic, and 7 (20%) patients had unknown stroke etiology. Patients with cardioembolic stroke were substantially older, had no or insufficient oral anticoagulation at the time of the first and repeated LVO, and repeated LVO occurred very early in 50%. Seventeen (49%) patients had a NIHSS score of 0 or 1 at discharge and seven (20%) patients died in the hospital after repeated MT. No bleeding complications occurred.

**Conclusions:** Repeated MT due to recurrent LVO is a rarely performed, safe, and effective procedure in acute stroke patients. Missing or insufficient anticoagulation is the most frequent cause for recurrent LVO.

## Introduction

Mechanical thrombectomy (MT) is an evidence based treatment in acute stroke patients with large vessel occlusion (LVO) in the anterior brain circulation (1). It has been estimated that between 7 and 15% of all stroke patients could be candidates for MT (2, 3). However, data on rate, etiology and time-point of recurrent LVO after a first MT and outcome of repeated MT in such a scenario is scarce. To date, there are only a few case reports (4–7) and one retrospective study with 15 repeated MT due to recurrent LVO (8) which have addressed this issue. We therefore investigated time point, etiology and short-term outcome of repeated mechanical thrombectomy due to repeated LVO after a first MT with modern stent retrievers and thromboaspiration systems in a large tertiary neurovascular center.

## Methods

We retrospectively reviewed all acute stroke patients treated with MT using modern stent retrievers and thromboaspiration systems at our tertiary neurovascular center between January 2010 and end of June 2018 and identified all patients who received two or more MT treatments due to a recurrent LVO in the anterior or posterior brain circulation. All patients received control brain imaging with computed tomography (CT) or magnetic resonance imaging (MRI) at 20–30 h, and color coded duplex sonography of the extra- and intracranial brain supplying blood arteries at 24–48 h after MT procedure. Additional CT or MRI angiography of the brain supplying arteries was performed in case of neurological deterioration after MT. All patients were treated on a specialized stroke unit or intensive care unit for at least 48 h with continuous ECG monitoring to detect atrial fibrillation after the MT procedure. Patient charts were evaluated regarding demographics, cardiovascular risk factors (hypertension, hyperlipidemia, diabetes mellitus, current smoking), atrial fibrillation, time elapse between the first and subsequent MT, use of antiplatelets or anticoagulants before and after the first and subsequent MT and stroke etiology. CT or MR imaging of the brain and brain supplying arteries (CT- or MR-angiography before the MT, digital subtraction angiography [DSA] and color coded duplex sonography) was reviewed by the authors for stroke etiology, hemorrhagic complications, reperfusion status using the Thrombolysis in Cerebral Infarction (TICI) score, and to assess vessel patency after MT. Stroke severity was scored using the National Institute of Health Stroke Scale (NIHSS) on admission and at discharge. The ethics committee of the Chamber of Physician North Rhine, Germany, approved the study.

## Results

We identified 35 (1.4%) out of 2,470 acute ischemic stroke patients treated with MT who had two or more MT procedures due to recurrent LVO. Thirty-four patients were treated twice with MT and one patient three times. Mean age at the time of the first MT was 69.3 (± 15.8, range 28–86) years and 20 (57%) were female. The mean baseline NIHSS score at admission before the first MT was 12.6 ± 6.5 (range 1–31). Successful reperfusion (TICI score of 2b or 3) was achieved in all patients with the first MT procedure. None of the patients experienced an intracerebral hemorrhage on control imaging. The mean NIHSS score at discharge was 3.2 ± 4.6 (range 0–19). Twenty (57%) patients had a NIHSS score of 0 or 1 at discharge. The mean time interval between the first and second LVO was 147 ± 241 (range 0–872) days. The mean baseline NIHSS score at admission due to the second LVO was 12.8 ± 7.0 (range 2–32). Successful reperfusion (TICI score of 2b or 3) in repeated MT was achieved in 31/35 (89%) patients. Again, no intracerebral hemorrhage occurred. Seven (20%) of the 35 patients died after the repeated MT and the mean NIHSS score at discharge in the remaining 28 patients was 6.0 ± 8.0 (range 0–27). Seventeen (49%) of the 35 patients had a NIHSS score of 0 or 1 at discharge. Patient details are displayed in Table 1.

**Table 1:** Detailed patient and procedural data.

**Case**	**Age range**	**Time between procedures, days**	**Procedure**	**Site of vessel occlusion**	**Antiaggregation medication prior to mechanical thrombectomy**	**NIHSS at admission at discharge**	**Stroke etiology**
1	60–65	406	1st	M1 (R)	None	14 → 0	AE
			2nd	ACI + M1 (R)	Aspirin	12 → 16	
2	75–80	2	1st	M1 (L)	None	13 → 7	CE
			2nd	M1 (L)	Aspirin	12 → 16	
3	70–75	1	1st	M1 (R)	None	16 → 2	CE
			2nd	M1 (R)	Aspirin	15 → death	
4	80–85	95	1st	M2 (L)	Aspirin	13 → 1	Undetermined
			2nd	M2 (L) + A2	Aspirin	6 → 3	
5	80–85	2	1st	M1 (L)	None	19 → 19	CE
			2nd	BA	Aspirin	30 → 19	
6	85–90	42	1st	M1 (R)	None	15 → 0	CE
			2nd	M1 (R)	Clopidogrel	9 → 9	
7	55–60	115	1st	M2 (L)	VKA (INR 1.7)	7 → 0	CE
			2nd	M2 (L)	None	6 → 1	
8	70–75	490	1st	M1 (L)	None	10 → 1	CE
			2nd	M1 (R)	Aspirin	n.a.	
9	80–85	3	1st	ICA-T (L)	Aspirin	21 → 12	CE
			2nd	M1 (R)	Aspirin	25 → death	
10	80–85	15	1st	M1 (L)	Aspirin	11 → 2	CE
			2nd	M1 (R)	Aspirin	10 → 1	
11	60–65	5	1st	M1 (L)	None	15 → 2	LA
			2nd	M2 (L)	Aspirin	17 → death	
12	40–45	17	1st	M1 (R)	Aspirin	20 → 1	AE
			2nd	M1 (R)	Aspirin	8 → 0	
13	80–85	7	1st	M2 (L)	Aspirin	9 → 4	CE
			2nd	BA	Aspirin	31 → death	
14	75–80	17	1st	ICA (L) + M1 (L)	Aspirin	8 → 7	1st MT: AE
			2nd	ICA (L)	Aspirin + Clopidogrel	15 → 27	2nd MT: CE
15	75–80	872	1st	M1 (R)	None	7 → 0	CE
			2nd	Distal ICA (R)	Aspirin	11 → 1	
16	75–80	20	1st	ACI + M1 (R)	Aspirin	18 → 8	Undetermined
			2nd	M1 (L)	Aspirin	19 → 19	
17	35–40	20	1st	M1 (L)	None	12 → 5	Factor V Leiden mutation
			2nd	M1 (R)	Aspirin	12 → 1	
18	35–40	719	1st	BA	Clopidogrel	30 → 14	AE
			2nd	V4 (L+R) + BA	Aspirin + Clopidogrel	6 → 2	
19	70–75	1	1st	M1 (R)	Aspirin + Clopidogrel	15 → 9	CE
			2nd	M1 (L)	Aspirin + Clopidogrel	9 → 10	
20	70–75	5	1st	M1 (L)	n.a.	20 → 4	undetermined
			2nd	M2 (R)	Aspirin	9 → 2	
21	75–80	246	1st	ICA-T (R)	Aspirin	15 → 0	undetermined
			2nd	ICA-T (L)	Aspirin	20 → death	
22	80–85	612	1st	M2 (L)	VKA (INR 1.4)	12 → 1	CE
			2nd	A1 (L)	VKA (INR 2.7)	12 6	
23	80–85	575	1st	M1 (R)	VKA (INR 1.7)	6 → 0	1st MT: CE
			2nd	CCA (L) + M2 (L)	VKA (INR 1.9)	12 → 3	2nd MT: AE
		2	3rd	A1 (L) + M3 (L)	Aspirin	3 → death	3rd MT: CE
24	70–75	186	1st	M1 (L)	Aspirin	21 → 0	Undetermined
			2nd	M1 (R)	Aspirin + Clopidogrel	9 → 0	
25	70–75	357	1st	AV (R)	VKA (INR 4.5)	2 → 0	1st MT: AE
			2nd	M2 (L)	VKA (INR 1.2)	7 → 0	2nd MT: CE
26	60–65	5	1st	SCA (R)	None	1 → 1	AE
			2nd	BA	Aspirin + Clopidogrel	30 → 5	
27	35–40	4	1st	ICA (L) + M1 (L)	None	18 → 7	Undetermined
			2nd	ICA-T (L)	Aspirin	14 → 3	
28	75–80	93	1st	M1 (R)	None	11 → 0	AE
			2nd	M1 (R)	DOAC	16 → 0	
29	75–80	2	1st	ICA (R) + M1 (R)	NMWH	12 → 3	CE
			2nd	M2 (L)	Aspirin	20 → death	
30	65–70	35	1st	ICA (L) + M1 (L)	None	11 → 0	AE
			2nd	CCA + ICA (L)	None	6 → 0	
31	55–60	18	1st	M1 (R)	None	14 → 0	AE
			2nd	M1 (R)	Aspirin	6 → 0	
32	75–80	5	1st	M1 (L)	NMWH	8 → 1	CE
			2nd	M1 (R)	DOAC	12 → 0	
33	75–80	0	1st	M1 (R)	Aspirin	15 → 0	CE
			2nd	M1 (L)		16 → 0	
34	25–30	0	1st	VA (L) + BA	None	2 → 0	AE
			2nd	BA	Aspirin	4 → 0	
35	70–75	15	1st	M1 (R)	Aspirin	1 → 0	Undetermined
			2nd	M1 (L)	Aspirin	11 → 20	

*Age range instead of the exact patient age is presented in order to minimize identifiable patient data*.

*NIHSS, National Institutes of Health Stroke Scale; MT, mechanical thrombectomy; M1, M1 segment of the middle cerebral artery; M2, M2 segment of the middle cerebral artery; A1, A1 segment of the anterior cerebral artery; CCA, common carotid artery; ICA, internal carotid artery, ICA-T, internal carotid artery terminus; SCA, superior cerebellar artery; VA, vertebral artery; BA, basilar artery; (R), right; (L), left; AE, arterioembolic; CE, cardioembolic; OAC, oral anticoagulation; VKA, vitamin K antagonist; INR, international normalized ratio; DOAC, direct oral anticoagulant; NMWH, non-molecular weight heparin; n.a., not assessed*.

Stroke etiology could be identified in 28 (80%) of the 35 patients with recurrent LVO receiving MT. A cardioembolic cause was found to cause the first and recurrent LVO in 15 (43%) patients (13 patients with atrial fibrillation, one patient with mechanical aortic valve, and one patient with ventricular cardiac thrombus after coronary artery bypass grafting). All 15 patients with cardioembolic stroke had no or insufficient oral anticoagulation (OAC) at the time of their first LVO stroke, while 13/15 of these patients did also have no or insufficient OAC at the time of their recurrent LVO stroke. Nine (26%) patients had an arterioembolic cause with recurrent LVO stroke in the same vessel territory: large-artery extra- (*n* = 4) or intracranial (*n* = 2) atherosclerotic stenosis, arterial dissection in 2 patients, and carotid artery web in 1 patient. Two patients received a carotid stenting in the session of the second MT and the vertebral artery was artificially closed in two other patients to prevent recurrent embolization. Carotid endarterectomy was performed in one patient after the second MT procedure. There were three (9%) patients with a mixed cardio- and arterioembolic stroke etiology of the first and recurrent LVO stroke, and stenting was performed in these patients. One patient with contralateral recurrent LVO had heterozygous factor V Leiden mutation. Stroke etiology could not be identified in 7 (20%) patients despite intensified evaluation after both the first and second LVO stroke including holter ECG recording for at least 3 days and transesophageal echocardiography. Five of these seven patients with embolic stroke of undetermined source had contralateral recurrent LVO suggesting a cardioembolic stroke cause.

The mean age of 78 ± 7 (range 57–86) years in the 15 patients with cardioembolic strokes was substantially higher compared to 55 ± 17 (range 28–76) years in the 9 patients with arterioembolic stroke. Seven (47%) of 15 recurrent LVO in patients with cardioembolic stroke occurred during the first 3 days after the first MT, while only 1/9 (11%) patients in the group with arterioembolic stroke had a very early recurrent LVO. The median time interval between the first and subsequent LVO stroke was 5 (range 0–872) days in cardioembolic and 18 (0–719) days in arterioembolic stroke.

## Discussion

Our retrospective case series is the largest study to date that investigated time point, etiology, and short-term outcome of repeated MT due to recurrent LVO stroke. Overall, repeated MT was performed rarely (1.4% of all MT procedures) in our large volume neurointerventional center. This finding was in line with another retrospective case series from a large neurointerventional center in the US (8). These authors reported that 15 (2%) of 697 patients with LVO stroke had repeated MT. However, it is likely that we have missed patients with recurrent LVO stroke after first MT as our study was not population based and several factors may have contributed to an underestimation of repeated MT. First, all but one patient in our study showed a considerable improvement of their baseline NIHSS after the first MT. Patients with no clinical improvement or no successful reperfusion after the first MT are probably more likely not be regarded as candidates for repeated MT in case of recurrent LVO. Second, stroke patients with recurrent LVO stroke might have presented to other stroke center in our metropolitan area.

Rates of successful recanalization were high both for the first and repeated MT procedures. There were no asymptomatic or symptomatic intracranial bleeding complications in LVO stroke patients treated with repeated MT, underlining again the safety and effectiveness of such a procedure. Not unexpected, early neurological improvement occurred slightly less frequently after second MT (47 vs. 57%) and in-hospital mortality rate was 20% after second MT. Bouslama et al. also reported that 20% of their stroke patients treated with repeated MT were deceased at 90 days (8). We are not able to report long-term functional outcome at a predefined time point in all patients (i.e., modified Rankin scale at 3 months), which is another limitation of our retrospective study.

Almost all LVO stroke patients with cardioembolic etiology had no or no sufficient OAC at the time of their first and recurrent LVO. Fifty percent of the recurrent cardioembolic LVO strokes occurred in the first 3 days after the successful initial MT procedure. This finding confirms the recommendation of subsequent stroke unit treatment of MT patients for at least 72 h to detect early LVO stroke recurrence as fast as possible (9). Furthermore, current guidelines do not provide evidence-based recommendations on optimal time-point to start OAC after acute ischemic stroke. All randomized trials with non-vitamin K antagonist oral anticoagulants start treatment at 14 days after the index stroke. Our data support the need to investigate whether OAC should be started earlier in acute stroke patients (10, 11).

Since five of the seven recurrent LVO in patients with unknown stroke etiology occurred contralateral to the first LVO, a cardioembolic stroke etiology (i.e., paroxysmal atrial fibrillation) is highly presumed, especially in patients older than 70 years. Patients with arterioembolic stroke were substantially younger and all recurrent LVO occurred in the same arterial vessel territory.

In conclusion, repeated MT in LVO stroke is a rare condition and can be performed safely with a high effectiveness. Cardioembolic stroke with no or no sufficient OAC was the most frequent cause for recurrent stroke due to LVO.

## Ethics Statement

The ethics committee of the Chamber of Physician North Rhine, Germany, approved the study.

## Author Contributions

RW and RC were involved in study design. RW, PS, and RC were involved in patient data and brain imaging analysis. RW was involved in gaining ethical approval and literature search. RW wrote the first draft of the manuscript and is guarantor. All authors reviewed and edited the manuscript and approved the final version of the manuscript.

### Conflict of Interest Statement

RW received speaker honoria from Medtronic and Bristol Myers Squibb, and from serving on a scientific advisory board of Medtronic. PS received consulting fee from Acandis and Covidien, speaker honoraria from Microvention, and proctoring agreement from Balt and Microvention. RC received consulting fee or speaker honoraria from Balt, Covidien, Microvention, Neuravi, Siemens and Stryker.
